# Evaluation of the Cost-Effectiveness of Pyramidal, Modified Pyramidal and Monoscreen Traps for the Control of the Tsetse Fly, *Glossina fuscipes fuscipes*, in Uganda

**DOI:** 10.1673/031.007.4701

**Published:** 2007-09-05

**Authors:** P.P. Abila, J. Okello-Onen, J.O. Okoth, G.O. Matete, F. Wamwiri, H. Politzar

**Affiliations:** ^1^ National Livestock Resources Research Institute P.O. Box 96 Tororo, Uganda; ^2^ Faculty of Science, Gulu University, P.O. Box 166, Gulu, Uganda; ^3^ Community Based Vector Control Organisation, P.O. Box 806, Tororo, Uganda; ^4^ Trypanosomiasis Research Centre, Kenya Agricultural Research Institute, P.O. Box 362, Kikuyu, Kenya; ^5^ Interafrican Bureau for Animal Resources, P.O. Box 30786, Nairobi, Kenya

## Abstract

Several trap designs have been used for sampling and control of the tsetse fly, *Glossina fuscipes fuscipes,* Newstead (Diptera: Glossinidae) based on preferences of individual researchers and program managers with little understanding of the comparative efficiency and cost-effectiveness of trap designs. This study was carried out to evaluate the cost-effectiveness of four commonly used trap designs: monoscreen, modified pyramidal and pyramidal, relative to the standard biconical trap. The study was performed under high tsetse challenge on Buvuma Island, Lake Victoria, Uganda, using a 4 × 4 Latin square design replicated 3 times, so as to separate the trap positions and day effects from the treatment effect. A total of 12 trap positions were tested over 4 days. The monoscreen trap caught significantly higher numbers of *G. f. fuscipes* (P<0.05) followed by biconical, modified pyramidal and pyramidal traps. Analysis of variance showed that treatment factor was a highly significant source of variation in the data. The index of increase in trap catches relative biconical were O.60 (pyramidal), 0.68 (modified pyramidal) and 1.25 (monoscreen). The monoscreen trap was cheaper (US$ 2.61) and required less material to construct than pyramidal trap (US$ 3.48), biconical and the modified pyramidal traps (US$ 4.06 each). Based on the number of flies caught per meter of material, the monoscreen trap proved to be the most cost-effective (232 flies/m) followed by the biconical trap (185 flies/m). The modified pyramidal and the pyramidal traps caught 112 and 125 flies/m, respectively.

## Introduction

**Figure 1.**
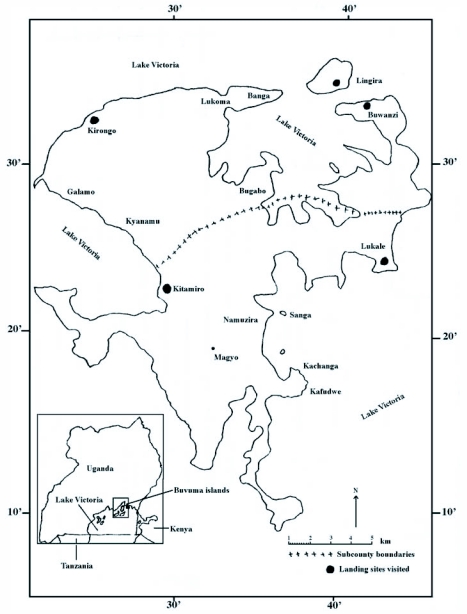
Map of Buvuma Island, Lake Victoria, where the experiments were conducted.

Traps and targets are a more acceptable means of controlling tsetse than ground or aerial spraying of insecticides in terms of the direct ecological and environmental impact control operations might have (Leak 1999). Trapping has been widely used as a basic sampling and control technique in tsetse control programes (Wall and Langley 1991). The development of this method has concentrated on improved and cheaper designs of the target in order to attract as many tsetse flies as possible and increase the number of tsetse actually landing on a target (Leak 1999). This would allow fewer targets to be deployed per unit area, hence reduce costs (WHO 1998). However, the attraction of flies to traps is influenced by a number of factors including activity, physiological state (nutrition, pregnancy/sex), season, sampling method, weather, time of day, visibility and vegetation (Harley 1958).

It is widely believed that successful control of tsetse should involve the local community, which are the intended beneficiaries (Leak 1999). Community ownership of a project is the only way to ensure sustainability. Several attempts have been made to carry out tsetse control with community participation, with varying problems with sustainability (Dransfield et al. 1991; Lancien 1991).

**Figure 2.**
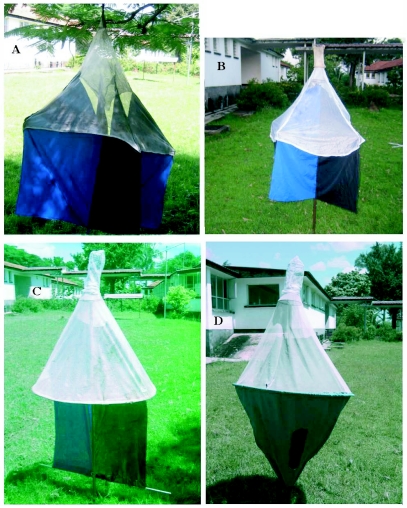
Photographs of the different trap designs that were tested. A) Pyramidal trap, B) Modified pyramidal trap, C) Monoscreen trap, D) Biconical trap.

In Uganda, a number of trap designs have been used for tsetse sampling and control, based on individual preferences of researchers and program managers. Lancien (1991) used impregnated pyramidal traps (Gouteux and Lancien 1986) and successfully controlled *Glossina fuscipes fuscipes* Newstead (Diptera: Glossinidae) and trypanosomosis in south-eastern Uganda. Okoth et. al. 1991 also involved the rural community in the use of non-impregnated monoscreen traps and was able to reduce fly catches to undetectable levels, but the tsetse fly populations in both test areas soon recovered.

The choice of which trap design to use should be based on reliable data to weigh the efficiency, cost-effectiveness and simplicity in design, ease of setting up and maintenance. The comparative efficiency and cost-effectiveness of different traps for the control of the various species of tsetse is so far little understood. This study was carried out to evaluate the cost-effectiveness of pyramidal, modified pyramidal and monoscreen traps for the control of *G.f.fuscipes* in south-eastern Uganda.

**Table 1.**
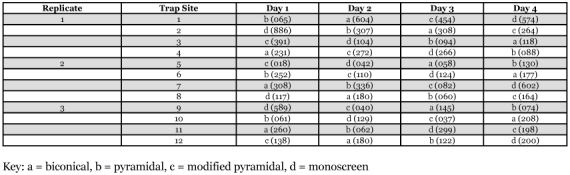
The catches of female *Glossina fuscipes fuscipes* in different trap designs by trap sites and replicates.

## Materials and Methods

### Study Area

The experiment was carried out on Buvuma Island (33°12′E to 33°25′E and 0°5′N to 0°20′) in Lake Victoria, Mukono District, Uganda in October 2001. The experimental sites were in Banga, Lukoma and Kirongo (Figure 1) along the shores where the population density of *G. f. fuscipes* was very high (Ogwal and Kangwagye 1988). These areas are characterised by riparian vegetation, moist evergreen forest with permanent papyrus forest swamps (Landale-Brown et al. 1964). The major grass species included *Imperata cylindrica, Hyperenia* species and sedges. The large trees included mangoes *(Mangifera indica), Maesopsis emini, Albizia* species, *Combretum* species, *Polyscias* and *Acacia* species (Eggeling and Dale 1951; Lind and Tallantire 1962). Rainfall is bimodal being high in March-July and September-November, interspersed by short dry seasons.

**Table 2.**
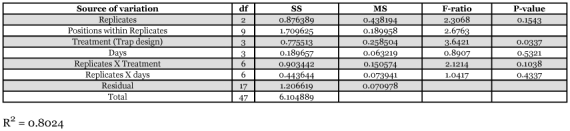
Analysis of variance (ANOVA) of female *Glossina fuscipes fuscipes* caught in different trap designs.

### Experimental traps

The trap designs tested were pyramidal trap (Lancien and Gouteux 1987); modified pyramidal trap (Lancien 1991); biconical trap (Challier and Laveissiere 1973); and monoscreen trap (Okoth 1991). They were made at COCTU (Co-ordinating Office for the Control of Trypanosomiasis in Uganda), Entebbe from lightweight blue and black polyester materials supplied by Vestergaard Frandsen. No artificial or natural odour baits were used in this experiment.

### Experimental design

The performance of different trap designs were compared in 3 replicates of a 4 × 4 Latin square design, using 12 trap sites over 4 days, so as to separate the trap positions and day effects from the treatment effect. The trap positions were randomised prior to deployment. Traps were set at about 17.00 hours just after the evening peak of fly activity and harvested the following day after 17.00 hours. The traps were rotated everyday for 4 consecutive days to the next randomised position, so as to test each trap design at every site.

### Data Analysis

The data was disaggregated by sex, and the analysis was based on female tsetse flies. Data were subjected to a log (× +1) transformation prior to analysis of variance procedure to determine differences in trap catches. The Student-Newman-Keuls (SNK) multiple range test was used to determine the significant differences between treatment means.

**Table 3.**
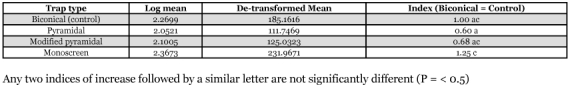
The indices of increase in trap catches and significant tests between treatment means.

## Results

### Trap Catches

A total of 15,598 flies were caught during the study, 33.58% males and 66.42% females, giving a sex ratio of 1:2. Analysis of variance showed that treatment factor was a highly significant source of variation in the data (Table 1).

Differences in numbers of female tsetse flies caught were significant between the different trap designs (Table 2). The monoscreen trap captured the highest number of flies followed by the biconical, modified pyramidal and pyramidal traps (P = 0.03). Tests between treatment means (using the Student-Newman-Keuls multiple range test) showed that the monoscreen trap caught significantly more female *G. f. fuscipes* than the pyramidal trap (P >0.05) but the catches between the modified pyramidal, pyramidal and biconical traps did not differ significantly (P >0.05) (Table 2).

### Indices of increase

The indices of increase in trap catches relative to biconical (control) clearly showed that the monoscreen trap was superior to the pyramidal trap, though it was not significantly superior to the biconical and modified pyramidal traps (P >0.05) (Table 3).

**Table 4.**
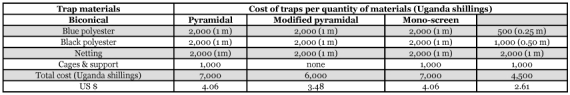
Estimated costs of monoscreen, biconical, pyramidal and modified pyramidal traps

### Cost-effectiveness of traps

The costs of different trap designs were computed on the basis of the quantity of materials used and their costs at the prevailing market price (Table 4). The monoscreen was the cheapest trap at 4,500 shillings (US$ 2.61) and required less material to construct. This was followed by pyramidal trap at the cost of 6,000 shillings (US$ 3.48). The biconical and the modified pyramidal traps had the highest cost at 7,000 (US$ 4.06) each.

**Figure 3.**
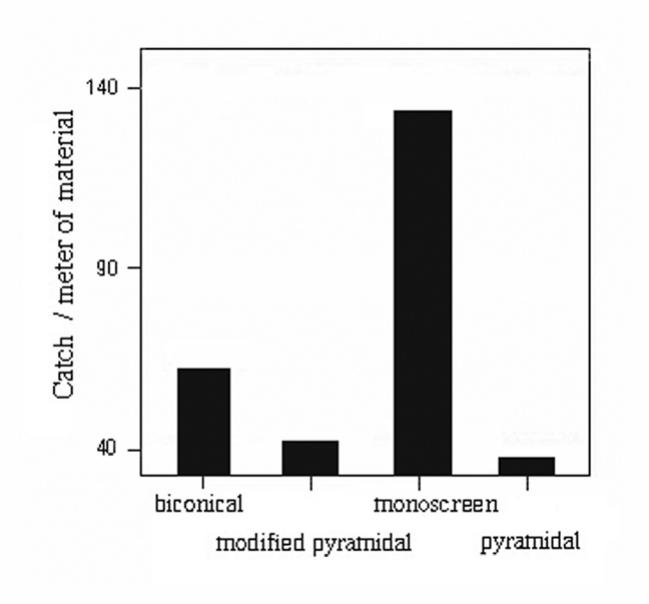
The number of female flies caught per meter of material used for each trap design.

Computation of the number of female flies caught per meter of material of each trap design is graphically presented in Figure 3. The result showed that the monoscreen trap was the most cost-effective (232 flies/m), followed by the biconical trap (185 flies/m). The modified pyramidal and the pyramidal caught 112 and 125 flies/m, respectively.

## Discussion

It is acknowledged that an understanding of tsetse population dynamics is essential for assessing tsetse control interventions and for understanding the epidemiology of human and animal trypanosomosis (Leak 1999). Such an understanding is facilitated by the attraction of flies to sampling techniques that differ in efficiency. In this study, the monoscreen trap proved to be more efficient in tsetse catch than the biconical, modified pyramidal and pyramidal traps. The trap caught significantly more tsetse flies and had the highest index of increase (1.25) than the other traps. Similar results were obtained by Okoth (1991), which suggests that the monoscreen trap is superior to the pyramidal trap that has been regarded as the standard trap for *G.* *f.fuscipes* control in Uganda. The lowest index of increase (0.6) registered by the pyramidal trap was over 10 fold that recorded by Okoth (1991). This difference may be attributed to the fact that the pyramidal traps we used were tailed and fastened to the ground so as to prevent excessive oscillation due to wind.

Comparison of the costs of different trap designs confirmed monoscreen as the cheapest trap that required the least quantity of materials to construct. The pyramidal trap does not require non-cloth materials and this is supposedly the reason it would appear more attractive, but the amount of cloth that the design takes evens out this advantage (Table 3). Moreover, the other traps would still be made more cost-effective by using typically available materials from forest resources to construct the support as described by Okoth (1991) in south-eastern Uganda. A successful community based sleeping sickness control program using the pyramidal traps in Southern Sudan was attributed to the simplicity of trap design, ease with which the traps were made, set up and maintained (Joja 2001); attributes which the monoscreen favourably shares.

Although estimation of the cost-effectiveness of traps is known to be difficult (Brightwell 1987), the monoscreen trap proved to be cost-effective in terms of the highest number of flies caught per meter of trap material. An earlier study reported that this trap took less material and was simpler to construct by rural communities using local plants (Okoth 1991). These attributes therefore make the monoscreen trap suitable for the control of *G.f.fuscipes.*
